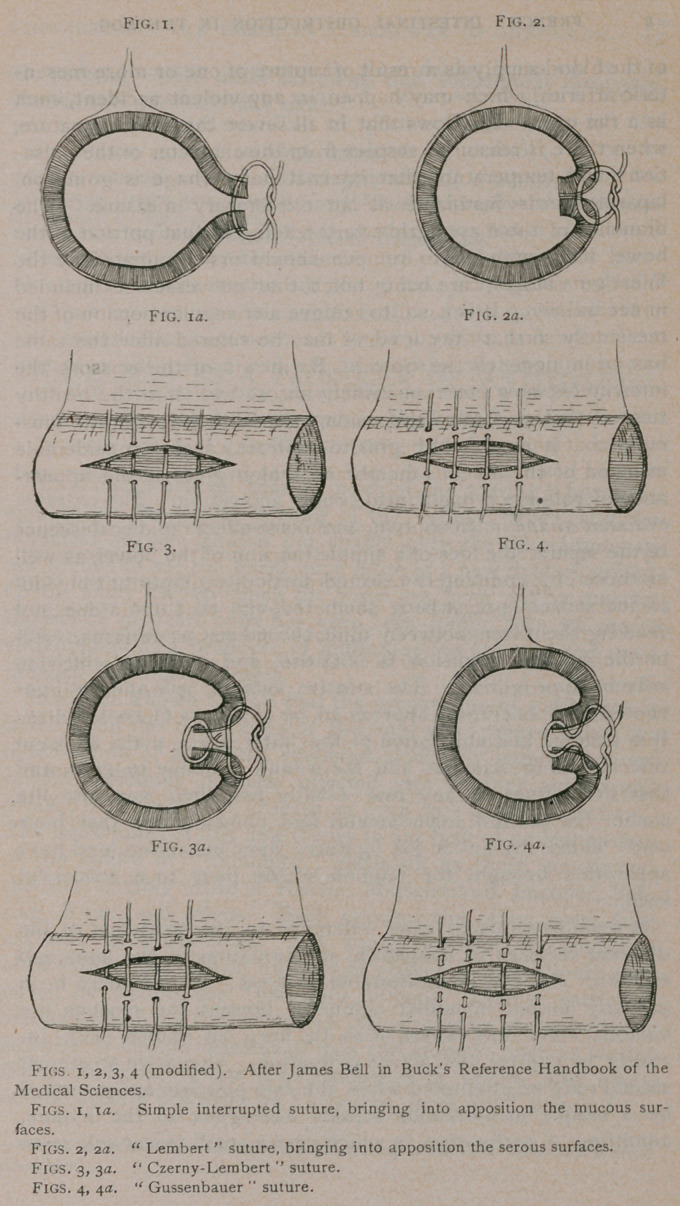# Intestinal Obstruction in the Dog

**Published:** 1896-01

**Authors:** Cecil French

**Affiliations:** The Washington (D. C.) Canine Infirmary


### THE JOURNAL
OF
### COMPARATIVE MEDICINE AND VETERINARY ARCHIVES.

Vol. XVII. *JANUARY*, 1896. No. 1.

### INTESTINAL OBSTRUCTION IN THE DOG: ITS TREATMENT, AND THE OPERATIONS OF LAPAROTOMY, ENTEROTOMY, ENTERECTOMY, AND ENTERORRHAPHY.

By CECIL FRENCH, D.V.S.,
THE WASHINGTON (D. C.) CANINE INFIRMARY.

The term "intestinal obstruction" includes all cases in which the onflow of the contents of the intestinal canal is obstructed. The less degrees of constipation do not, however, form part of the consideration.

Occlusion of the canal is sometimes congenital, most frequently in the region of the anus (*atresia ani*), where the integument remains imperforate or the rectum ends in a blind sac.

The classification of all other cases into three principal groups, adopted by the late George Ross, M.D., in an article on "Intestinal Obstruction in the Human Being,"[1] is equally applicable to the dog:

I. From compression, obliteration of the canal taking place from pressure without.

II. From obstruction, blocking of the canal by obstruction within its walls.

III. From constriction, obstruction by causes developing in connection with the wall itself.

1. Obstruction arising from compression without may be due to slits in the mesentery or omentum. These may follow care-

[1] Buck's Reference Handbook of the Medical Sciences.

165150

less laparotomy operations, more particularly after ovariotomy, where perforation of the broad ligament has taken place. Abnormal fibres and false ligaments left as a result of former peritonitis and the various herniæ are possible causative agents of constriction. Volvulus has been recorded occasionally in the journals, and is possible from very severe vomiting, to which dogs are so prone. A remarkable case of torsion of the stomach came to light at Munich, which was fully described by Kitt, in his *Monatshefte für praktische Thierheilkunde*, 1894. Intussusception, or inversion of one part of the intestine into a part immediately adjoining, is not uncommon, and appears to depend on irregular muscular action of the walls. Congestion of the vessels and inflammation follow invagination of a portion of the bowel, resulting in intermucous and interserous adhesions. Gangrene may ensue and sloughing of the entire inverted section take place, so that it is ultimately discharged at the anus.

Other causative factors to be considered are swelling of the prostate gland, abdominal abscesses, and ascites.[1]

2. Obstruction by foreign bodies. This is not at all uncommon in grown dogs from bones or fragments of bone which have failed to become digested during their sojourn in the stomach. It is questionable whether at any time bones constitute a proper food for dogs. Not only do they severely overtax the digestive apparatus, but they are liable to pass through into the intestine, to form there the nucleus of some fecal calculus. Subjects with gastritis will often swallow stones and other cold substances; while severe irritative eczemas, by causing an animal to tear and consume its hair, may lead to the formation of impacted hair-masses. Pet dogs given balls and similar articles to play with will swallow them accidentally or otherwise. The presence of plugs of matted ascarides are sufficient in themselves and often induce an abnormal appetite and craving for foreign substances, when buttons, peach-stones, cork-stoppers, marbles, nuts, tacks, or almost any foreign body, are liable to be picked up. Müller records the swallowing of a sponge. Such matters are probably swallowed before the self-education of the puppy in the matter of victuals is complete.

Obstruction by fecal masses (coprostasis) is a condition due

[1] Müller: Die Krankheiten des Hundes.

to paralysis of a section of the bowel. It is most common in the rectum, and is caused by undue solidity of the feces from the nature of the food, and (what is only too true of a large percentage of all canine diseases) want of exercise and overfeeding, with its resultant sluggish liver. Hemorrhoidal nodules are occasionally sufficient cause for the development of this trouble, depending in turn on the condition of the liver. Perineal hernia will also exert sufficient pressure on the rectum to produce occlusion.

The intestine at the proximal side of any obstruction manifests considerable dilatation and hypertrophy, the result of the abnormally active peristalsis by which the muscle has tried to force on the obstacle, a condition in striking contrast to the narrow, contracted, empty distal portion. The irritation produced by these masses may set up a subacute muco-enteritis. Ulceration of the wall is uncommon, the death of the animal from inanition, combined with collapse from copræmia, usually intervening before such a process can be established.

3. Obstruction from constriction. This includes all forms of neoplasms and cicatrices. A transverse wound occurring during crude abdominal operations when cicatrized, or even stricture following coalescence of the resected bowel after the operation of enterectomy, may completely occlude the tube.

Since the effects of intestinal obstruction develop chiefly from causes of a purely mechanical nature, the various types of this trouble are so very similar that the symptoms may be described in common.

These usually come on suddenly and are vague. As in all abdominal troubles of the dog, reflex vomiting is very pronounced and constant, and is greatly influenced by the position of the obstruction and the degree of medication that has been resorted to. If the obstruction occur in or near the duodenal region, and nematodes be present, the extreme degree of muscular contraction induced by it is likely to cause the latter to migrate to the stomach and be thence forcibly ejected with the vomitus, which is usually deeply bile-stained, owing to the tendency the act of vomiting exerts in causing expulsion of the contents of the gall-bladder and bile-ducts. This occurrence is apt to lead to a false diagnosis of stomachic and intestinal parasitism. If it be in the lower bowel, the vomitus will become offensively feculent, consisting of matter driven back into the stomach by antiperistalsis. The ileum, having the smallest lumen of any portion of the intestine, is most frequently the seat of obstruction by foreign bodies. Such matters may be primarily arrested in their passage through upper parts of the bowel and induce the symptoms of obstruction, such hindrance being overcome by favorable conditions, only, however, to be permanently established lower down in the ileum. Constipation is complete, but a large portion of any feces on the distal side of the obstruction may be brought away by enemata. Diarrhœa may occur when the obstruction is near the gastric region. Blood may also be present in the alvine discharges. Pain is seldom very apparent, at least it is not manifested, nor is there much, if any, flatulent distention. Exceptions to this are witnessed in cases where the obstruction has either located itself or moved as far as the rectum, when great straining accompanied by piteous moaning indicates that the futile attempts at defecation are accompanied by distressing, hurtful sensations. These severe ineffectual expulsive efforts are attended by the passage of mucus or blood-stained mucus. Neither is vomiting in these cases such a prominent symptom, but the peculiar loss of motor and sensory power in the hind limbs (reflex paraplegia) is frequently seen. The etiology of the latter is still a matter of doubt, but the hypotheses of Leyden and Brown-Séquard are worthy of note. According to the former, the paralysis is to be explained by an ascending neuritis arising from the organs originally affected. Rosenbach, however, has demonstrated that if the wound in a traumatic neuritis remain aseptic, an extension of inflammation above the point of injury does not occur. Since we are not dealing with any traumatism, the above explanation is hardly satisfactory. On the other hand, the degree of pressure from the obstacle may be sufficient to bring about a mechanical destruction of the nervous elements in the intestinal walls, degeneration of the same, increase of connective tissue, and finally restoration.[1] The substance of Brown-Séquard's suggestion is that impulses travelling from the seat of irritation induce a hyper-excitability of the vaso-contractor centres and nerves controlling the arterioles of either the cord, efferent nerves, or certain groups of muscles, resulting in anæmia and starvation of one or all.

Another explanation is that a reflex inhibition is excited in certain motor areas by sensory irritation arising in the affected

[1] Strümpell: Text-book of Medicine.

part.[1] The presence of parasitic masses is usually accompanied by much meteorism, and, in the aforementioned case of stomach torsion, acute pain and tympanites were prominent symptoms. There is entire refusal of nourishment, the patient strenuously objecting to forced imbibition, anything thus given being invariably shortly regurgitated. A little water may be taken. The abdomen is usually drawn and hard, the intestines being gathered into the epigastric region, giving it a distended appearance. The expression is intensely dejected, the extremities cold, the pulse very small and rapid, and the temperature mostly falls. There are occasional nervous tremors, and the whole appearance and actions are suggestive that the animal wishes only to be left in peace that it may lie down and calmly await the end. If the obstruction be low down and some time in position, external abdominal pressure will cause local pain, even if the object cannot be distinctly felt.

In cases of acute obstruction the course of the disease may run on for a week or two, but in those developing slowly from constriction occlusion is gradual and the duration considerably longer, though sudden blocking may take place. The majority of cases of intestinal obstruction in the dog, unless relieved speedily by surgical procedure, terminate fatally. However, those from rectal impactions are more capable of recovery.

*Treatment.* As soon as prominent symptoms of intestinal obstruction have been recognized, an examination of the rectum by means of some long blunt instrument should be made in order to ascertain if coprostasis be present. By sounding in this manner a pretty accurate idea of the consistency of the feces may be obtained, and the exceedingly unpleasant digital process obviated. The abdomen is then to be examined at all points where herniæ could possibly exist. Physical examination of the abdomen, especially in lean dogs, often gives valuable information. Should intussusception be the cause of the trouble careful palpation may reveal the tumefied invaginated layers of bowel. Where foreign bodies, such as balls, etc., form the obstruction, it is often possible in this way to definitely decide their nature.

All obstruction cases call for prompt and attentive treatment. In cases of rectal obstruction enemata of equal parts of glycerin and warm water, soapsuds, or oil, containing one drachm of

[1] Ibid.

spirits of turpentine to the pint, frequently prove beneficial. When a patient can be made to submit, a continuous stream of warm water if persisted in for several minutes will soften and break up the hardest masses. If the stomach will bear it, which is commonly not the case, purgatives may be administered, but they are so liable to produce nausea and vomiting that we should rely mainly on operative measures to afford relief. As much of the mass as possible is to be removed by such instruments as one section of the bitch obstetric-forceps in the case of a large dog, or the handle of a spoon in that of a small animal. As soon as the rectum has been rendered permeable a quantity of semifluid feces is generally passed. After such measures, which are more or less irritating to the mucous membrane, it is well to inject some disinfectant, a two per cent. solution of creolin admirably answering the purpose, its value depending on its formation of a slippery coating over the parts.

It is little more than barely possible to relieve acute strangulated external herniæ by taxis; moreover, the immediate danger being too great to allow us to be dilatory in operative interference, the various methods for the surgical reduction of the same should be resorted to, for descriptions of which the reader is referred to the text-books. In turning to the older authors for the treatment of acute obstruction other than that of a fecal nature we find scarcely any advocation of operative procedure; indeed, one prominent teacher asserts his opinion that an operation would be as bad as the lesion. Remedies calculated to relieve spasm, purgatives, opium, etc., are recommended, the counsellors of this method of treatment being evidently forgetful that the effects of intestinal obstruction form the severest and most intractable conditions known to canine pathology. The highly excitable stomach of the dog becomes actively engaged in expelling the putrid contents of the intestine which have been forced backward into it, and is equally responsive to the irritation of foreign bodies introduced in the shape of drugs. In fact, in nearly every case it is perfectly useless to attempt any medication per orem at all. Stimulants, however, such as ether, trinitrin, caffein, may be hypodermically injected with advantage. Where the obstruction is beyond the reach of rectal interference and vomiting precludes the possibility of administering remedies the surgeon should not hesitate to operate immediately. No good comes of waiting, early operations offering much better chance of recovery. It is only after the inflammatory changes at the seat of lesion have developed into gangrene that the percentage of recovery is reduced to a minimum, but even then, by excision of the mortifying portion, life may be saved. With our modern antiseptic surgery no dog should be allowed to die without an attempt being made to render the canal permeable. We must remember that it is only by inducing a return of normal peristalsis, when a foreign body has once become lodged at any point of the intestinal canal, that we can hope for its removal. Should even a slight inflammatory process have started any further peristaltic action is at once checked. Every decided inflammation renders the muscular layers œdematous, and thus impedes their activity. In cases where an unduly large body has become deposited the muscular coat is so stretched as to become still further incapacitated for its proper functions.

We have here, therefore, additional factors that warrant surgical interference. The differential diagnosis of the various forms of obstruction without a history is, of course, usually an impossibility, but if the practitioner can reasonably suspect the existence of one form or other the immediate performance of laparotomy as an exploratory measure in all serious cases is justifiable. All cases of volvulus, intussusception, neoplasms, and cicatrices call for laparotomy, with the necessary supplemental operations demanded by the exigencies of each particular case. Should the two former conditions be irreducible by simple measures, they, with the two latter, require the operation of enterectomy. Enterotomy is applicable only to cases of obstruction by foreign bodies and gunshot-wounds.

*Laparotomy* should be performed under the strictest antiseptic conditions, instruments, sponges, the hands of operator and assistant alike being thoroughly disinfected. It is best to cleanse the skin in the immediate vicinity of the contemplated incision by warm water and soap and some powerful antiseptic solution, as well as to shave it of its hair. A sufficient number of threaded gut ligatures should be prepared and laid handy, care being taken that they are particularly well disinfected. The lateral incision through the abdominal wall, while not so convenient for exploratory purposes as the median one, is, after a careful consideration of the pros and cons of each, undoubtedly the more preferable in the dog. There need be little bleeding, especially if the muscular tissue is teased apart. The great objection to median incisions lies in the danger of the dissected parts failing to become completely united. The wall being least vascular in this region, and having to bear the pressure exerted by the pendent coils of intestine, which pressure is materially increased at each inspiration, the sutures are apt to give way before the healing process is firmly established, and a portion of the bowel protruding by gravitation through an aperture thus framed tends to form a hernia. This danger is almost entirely obviated by the lateral incision, the animal invariably lying with the wounded side uppermost. The incision through the integument may be on either side and should be made a short distance from the borders of the false ribs in a vertical or slightly oblique direction downward and forward, such a one admitting of subsequent unobstructed drainage. This position of lateral section is most satisfactory, inasmuch as it places all parts of the abdominal cavity within easy reach for exploration. The incision should first be long enough to allow admission of the thumb and one finger, and, if found necessary, can be enlarged later in either direction. In cases of large animals it will generally be found unavoidable to make such a wound that the whole hand may be allowed to enter the cavity. The subcutaneous tissue and muscular layers are then either successively divided in the same direction or by a method which prevents any great degree of hemorrhage, viz., teasing the fibres of each individual coat apart, and according to the direction in which their course lies. It is well to have an assistant with a small tenaculum handy, with which to hold back the divided parts, more especially if the subject for operation be a member of the smaller breeds. All bleeding having been arrested, the omentum is to be gently pushed aside, and unless the position of the obstruction has been located through the wall prior to the commencement of the operation, the operator will now make a systematic digital exploration, beginning in the pelvic region. The bowel on the distal side of an obstruction is usually collapsed, and if any portion in that condition be found it is to be withdrawn outside the cavity and dexterously passed through the fingers and returned until the seat of lesion is reached, any operation being continued outside the cavity. Extrusion of the bowel is to be prevented as much as possible, but if such incidentally take place it should be protected by envelopment in a few layers of warm sterilized gauze, which must be kept at an equable temperature as nearly as possible. The obstruction being found, the condition of the tissues in the immediate neighborhood is to be carefully noted, and according as to whether a gangrenescent condition has developed or not will depend the necessity of simple incision or excision of a part.

In closing the wound the edges of the muscular coats may be stitched by either gut or silk ligatures, preferably the former if the antisepsis is known to be thorough, as the ends may be cut off close and do not need to be left hanging out of the external wound, as do the latter. The skin is loosely united by silk sutures, in order to leave passage for the subsequent drainage. An antiseptic dressing should then be applied and a moderately tight bandage bound round the abdomen and kept on for a few days, in order to prevent the patient from attempting to tear open the wound before adhesion has taken place, the dressing being repeated twice daily.

*Enterotomy* may be performed for the relief of obstruction by any foreign body, provided the inflammatory process that the object has induced is not of such intensity that gangrene is threatened, when enterectomy is the only hope of a cure. The incision through the intestinal wall should be made longitudinally and in a lateral position midway between the lesser and greater curvatures. A circularly inclined incision when cicatrized might lead to dangerous stenosis. The intestine being suspended from the roof of the cavity, it will be readily understood that such an incision when sutured will not so readily permit of penetration by fecal fluids as one situated inferiorly on the greater curvature. The obstructing body being removed, the operator should gently compress the bowel between the thumb and first finger for a short distance above and toward the seat of lesion so that any fecal matter may be expelled, care being taken that none of it enter the cavity. The parts are then to be thoroughly washed with a warm disinfectant solution, sutured, returned to the cavity, the omentum replaced as near as possible in its normal position, and the wall closed.

*Enterectomy.* As already stated, the indications for this operation are irreducible volvulus and intussusception, neoplasms, cicatrices, and local gangrene, whether from inflammatory changes occurring as a result of an obstruction or from arrest of the blood-supply as a result of rupture of one or more mesenteric arteries, which may happen in any violent accident, such as a run-over. It follows that in all severe cases of this nature, when there is reason to suspect from the character of the pulsations and temperature that internal hemorrhage is going on, laparotomy is justifiable as an exploratory measure. The branches of the mesenteric artery leading to that portion of the bowel it is intended to remove should first be ligated by the finest gut sutures, care being taken that no vessel be included unnecessarily. It is usual to remove a triangular portion of the mesentery so that the cut edges may be sutured after the same has been done to the bowel. By means of the scissors the intestine is now cut transversely across well into the healthy tissue on either side of the lesion. On cutting across, the muscular coat immediately begins to contract, causing considerable eversion of the mucous membrane, which gives it the appearance of rolling on itself inside out.

*Enterorrhaphy*. In applying sutures to effect rapid coalescence of the separated edges of a simple incision of the bowel, as well as those of a completely resected portion, an important physiological principle must be remembered, viz., that union does not readily take place between mucous and serous surfaces. Accordingly, the mucous surfaces at the edge of a simple incision may be approximated and held in position by ordinary interrupted sutures (Figs. 1, 1*a*), which should be of fine sterilized gut, inserted at comparatively short intervals, and the ends cut off close to the knot. Or a more improved method of suturing is that bearing the name of "Lembert" (Figs. 2, 2*a*), with its modifications known as the Czerny-Lembert (Figs. 3, 3*a*) and "Gussenbauer" (Figs. 4, 4*a*). The first of these is theoretically more correct for simple longitudinal incision than the ordinary suture above referred to, for the reason that in the latter the serous, muscular, and mucous coats are pierced, thus allowing a possible passage for egress of intestinal contents and bacteria, whereas in the former the suture is passed through the serous and muscular coats only, leaving the mucous layers intact. The Czerny-Lembert and Gussenbauer sutures are both used for enterectomy operations, but are so irksome and complex that they are far better superseded by simpler measures. Moreover, the edges of the divided wall become, when approximated by that method, turned considerably inward toward the long

FIG. 1.

FIG. 2.

FIG. 1*a*.

FIG. 2*a*.

FIG 3.

FIG. 4.

FIG. 3*a*.

FIG. 4*a*.

FIGS. 1, 2, 3, 4 (modified). After James Bell in Buck's Reference Handbook of the Medical Sciences.

FIGS. 1, 1*a*. Simple interrupted suture, bringing into apposition the mucous surfaces.

FIGS. 2, 2*a*. " Lembert " suture, bringing into apposition the serous surfaces.

FIGS. 3, 3*a*. " Czerny-Lembert " suture.

FIGS. 4, 4*a*. " Gussenbauer " suture.

axis of the canal, which must, of necessity, seriously reduce its calibre, if only temporarily. Various supports have been placed into the canal experimentally to facilitate the insertion of the sutures. Cylinders of sterilized gelatin, turnip, potato, etc., and more recently the Murphy button for the human subject have been introduced, which latter, however, on account of its dimensions is not applicable to the dog, the smallest size made being serviceable only for members of the larger varieties. Whether a support is used or not, the sutures should be inserted in the following order, so that ridges and corrugations may not result: the first at the level of the mesenteric attachment, the second immediately opposite at the free border, the next two midway between these on either side, and so on until it is considered that perfectly tight juxtaposition of the parts has been secured. It is with the greatest difficulty that the parts of a resected bowel can be adjusted so that mucous, muscular, and serous coats are brought into proper apposition. The extreme eversion of the mucous coat and contractions of the muscular layers render the desired apposition almost an impossibility. However, by inserting the sutures moderately close to the edge of the serous coat, passing them through all three coats and drawing them comparatively tight, the nearest possible approach to the desired condition is obtained, and union by adhesive inflammation follows. The relative value of silk and gut ligatures in this operation appears to be a matter of controversy. It is claimed that absorption of the latter takes place so soon that the loop slackens and the wound is liable to gape, and that the former in any case usually find their way into the canal. In the few experimental and clinical cases that have come under my notice gut ligatures have been used, and have apparently brought the reunion of the parts to a successful issue.

It remains to make some reference to the treatment of the different varieties of congenital malformation of the anus and rectum. These it is undesirable to treat. Unless there be a properly formed muscular sphincter present the reasons are obvious. No one would wish to keep an animal with inability to control the act of defecation. Operative treatment should only be attempted when the two pouches are separated by a simple membranous septum analogous to the hymen, admitting of perforation, in which case a small trocar will serve the purpose. Care must be taken that subsequent cicatricial contraction does not take place.

REFERENCES.

Vickery and Knapp's translation of Strümpell's Text-book of Medicine.

Articles by George Ross, James Bell, and Francis Shepherd in Buck's Reference Handbook of the Medical Sciences.

Fröhner and Kitt's Monatshefte für praktische Thierheilkunde.

Müller: Die Krankheiten des Hundes.

## Figures and Tables

**Fig. 1. Fig. 2. Fig. 1a. Fig. 2a. Fig. 3. Fig. 4. Fig. 3a. Fig. 4a. f1:**